# Preoperative Serum Interleukin-6 Is a Potential Prognostic Factor for Colorectal Cancer, including Stage II Patients

**DOI:** 10.1155/2016/9701574

**Published:** 2015-12-27

**Authors:** Kazuyoshi Shiga, Masayasu Hara, Takaya Nagasaki, Takafumi Sato, Hiroki Takahashi, Mikinori Sato, Hiromitsu Takeyama

**Affiliations:** Department of Gastroenterological Surgery, Nagoya City University, 1 Kawasumi, Mizuho-cho, Mizuho-ku, Nagoya, Aichi 467-8601, Japan

## Abstract

*Aims*. To evaluate the prognostic significance of serum interleukin-6 (IL-6) in colorectal cancer (CRC). *Patients and Methods*. Preoperative serum IL-6 was measured in 233 CRC patients and 13 healthy controls. Relationships between IL-6 and various clinicopathological factors were evaluated, and the overall survival (OS) and disease-free survival (DFS) rates according to IL-6 status were calculated for all patients and according to disease stage. *Results*. The mean IL-6 level was 6.6 pg/mL in CRC patients and 2.6 pg/mL in healthy controls. Using a cutoff of 6.3 pg/mL, obtained using receiver operating characteristic curve analysis, 57 patients had a high IL-6 level. The mean value was higher for stage II disease than for stage III disease. IL-6 status correlated with C-reactive protein (CRP) and carcinoembryonic antigen levels, obstruction, and pT4 disease. The OS differed according to the IL-6 status for all patients, whereas the DFS differed for all patients and for those with stage II disease. The Cox proportional hazards model showed that pT4 disease was an independent risk factor for recurrence in all CRC patients; IL-6, CRP, and pT4 were significant risk factors in stage II patients. *Conclusions*. The preoperative IL-6 level influences the risk of CRC recurrence.

## 1. Introduction

Colorectal cancer (CRC) is the third most common cancer in the world. The curability of CRC has been improving in recent years, due to the progress made in surgical techniques and in chemotherapy, including molecular targeted drugs. However, the prognostic risk factors for CRC are still under debate. Recently, several biomarkers have been reported to be the risk factors of CRC recurrence; however, lymphatic metastasis, which Dukes discovered more than 80 years ago [1], remains the most useful predictive factor. On the other hand, a relationship between inflammation and poor cancer prognosis has been recently reported [2–5]. McMillan et al. demonstrated that high preoperative levels of C-reactive protein (CRP) and low preoperative levels of albumin were associated with a poorer prognosis after curative intensive resection of CRC [6]. A high CRP level is a characteristic feature of inflammation and, based on this result, many other inflammatory cytokines have been evaluated. Interleukin-6 (IL-6), one of the major inflammatory cytokines, has been investigated in various types of diseases. IL-6 is a multifunctional cytokine produced by various types of cells, including monocytes, macrophages, and fibroblasts. In addition, recent studies have revealed that certain cancer cells can also produce IL-6 and that serum IL-6 levels correlate with the progression of several different carcinomas, such as gastric, esophageal squamous cell, lung, prostate, and breast cancers, as well as CRC [7–12].

However, the risk of recurrence in CRC patients with node-negative disease is not clear. Accordingly, we aimed in this study to investigate the correlations between preoperative IL-6 levels and various clinicopathological parameters and to determine whether preoperative IL-6 is a prognostic factor for recurrence in CRC patients, with particular focus on stage II CRC.

## 2. Materials and Methods

### 2.1. Patients

Two hundred thirty-three patients with CRC were enrolled in this study. Written informed consent to participate was provided by all patients. Operations were performed between April 2010 and November 2013. The male : female ratio was 139 : 94, and the mean age was 69.1 years (±10.2). The mean body mass index (BMI) was 22.2 kg/m^2^ (±3.8). The exclusion criteria included the presence of a second cancer or the presence of inflammatory conditions such as abscess, perforation, or pneumonia. The primary lesion was located in the rectum in 78 patients and in the colon in 155 (sigmoid colon: 65, descending colon: 11, transverse colon: 26, ascending colon: 42, and cecum: 11). The levels of biomarkers (serum IL-6, CRP, albumin, carcinoembryonic antigen (CEA), carbohydrate antigen 19-9 (CA19-9)), and plasma vascular endothelial growth factor (VEGF) were measured preoperatively. Serum IL-6 level was measured using a standard chemiluminescent enzyme immunoassay, and plasma VEGF level was measured using an enzyme-linked immunosorbent assay, as suggested by the manufacturer (SRL Tokyo Laboratories Inc., Tokyo, Japan). CEA and CA19-9 levels were measured using standard chemiluminescent enzyme immunoassays. CRP level was measured using the latex agglutination reaction method. Albumin level was measured using the modified bromocresol purple method. Synchronous metastasis was evaluated using preoperative computed tomography (CT) and/or postoperative pathological examinations. According to the TNM classification, 13/233 (5.6%), 52/233 (22.3%), 60/233 (25.8%), 63/233 (27.0%), and 45/233 (19.3%) cases were classified as stages 0, I, II, III, and IV, respectively. In 32 patients, liver metastasis was observed, and 10 patients were discovered to have lung metastasis during the primary operation. All patients underwent surgery. Among them, 207 (all 188 stages I–III patients and 19/45 stage IV patients) underwent curative resection, while 26 of the 45 stage IV patients underwent palliative resection.

The correlations between preoperative serum IL-6 and the patient characteristics (age, gender, and BMI), preoperative blood test findings (serum albumin, serum CRP, serum CEA, serum CA19-9, and plasma VEGF), and tumor characteristics (tumor location, differentiation, degree of lymphatic and venous invasion, TNM classification, and obstruction) were analyzed (Table 1). The TNM stage was classified depending on the depth of tumor invasion (T), lymph node metastasis (N), and distant metastasis (M). Colorectal obstruction was defined as a condition requiring preoperative decompression or fasting. Postoperative follow-up evaluations were performed with serum CEA analysis and CT scans every 3 to 6 months. The mean follow-up period was 887 (±445.9) days.

### 2.2. Statistical Analysis

All statistical analyses were performed with EZR (Saitama Medical Center, Jichi Medical University, Saitama, Japan), which is a graphical user interface for R (The R Foundation for Statistical Computing, Vienna, Austria). More precisely, it is a modified version of the R-commander designed to add statistical functions frequently used in biostatistics [13]. Univariate analyses were performed using Fisher's exact test, and multivariate Cox logistic regression analysis was conducted to determine the independent prognostic factors. The overall survival (OS) and disease-free survival (DFS) rates were evaluated using Kaplan-Meier curves from all 233 patients and from the 207 patients who underwent curative intent resection, respectively. Statistical significance was defined as *P* < 0.05.

## 3. Results

### 3.1. Measured Values of Serum and Plasma Biomarkers

**Figure 1:**
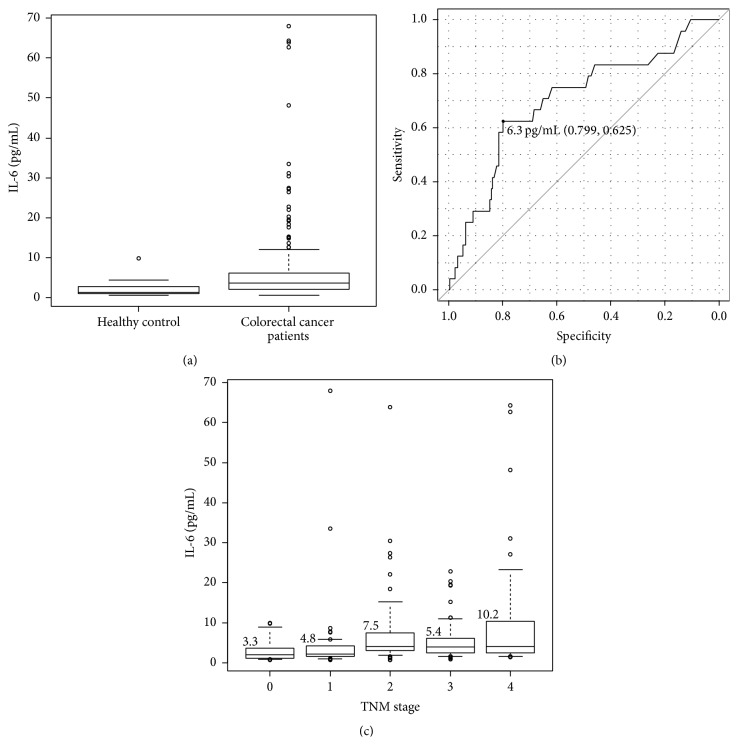
Measured serum interleukin-6 (IL-6) values. (a) Preoperative serum IL-6 values for all patients (*n* = 233). The mean value was 6.6 pg/mL (range: 0.7–68.0). (b) Receiver operating characteristic curve of IL-6 according to survival. The optimal cutoff was determined as 6.3 pg/mL. (c) IL-6 values according to the disease stage. Mean values: stage I: 4.8 pg/mL, stage II: 7.5 pg/mL, stage III: 5.4 pg/mL, and stage IV: 10.2  pg/mL.

The measured serum IL-6 values ranged from 0.7 to 68.0 pg/mL. The mean values were 6.6 (±9.9) pg/mL in CRC patients and 2.6 (±2.5) pg/mL in healthy controls (Figure 1(a)). Cancer-related death was considered as the endpoint, and the optimal cutoff value of serum IL-6 was calculated as 6.3 pg/mL using receiver operating characteristic curve analysis (Figure 1(b)). According to this cutoff value, 57/233 (24.5%) and 176/233 (75.5%) patients were classified as having elevated and normal IL-6 levels, respectively. The IL-6 values according to disease stage are shown in Figure 1(c). The mean value for each stage tended to differ: the mean value for stage II disease was higher than that for stage III disease (7.5 versus 5.4 pg/mL), although this difference was not significant.

Univariate analyses revealed that an increase in the IL-6 level was associated with low levels of albumin (<4.0 g/dL), high serum CRP (≥0.5 mg/L), high serum CEA (≥5.0 ng/dL), high serum CA19-9 (≥37 ng/dL), venous invasion, tumor depth of pT4 disease, distant metastasis, and colorectal obstruction (Table 2). Multivariate analysis revealed that high serum IL-6 independently correlated with high levels of CEA (*P* = 0.04), high levels of CRP (*P* < 0.001), pT4 disease (*P* = 0.04), and colorectal obstruction (*P* = 0.006) (Table 3).

### 3.2. Correlations with Patient Prognosis

#### 3.2.1. Overall Survival

**Figure 2:**
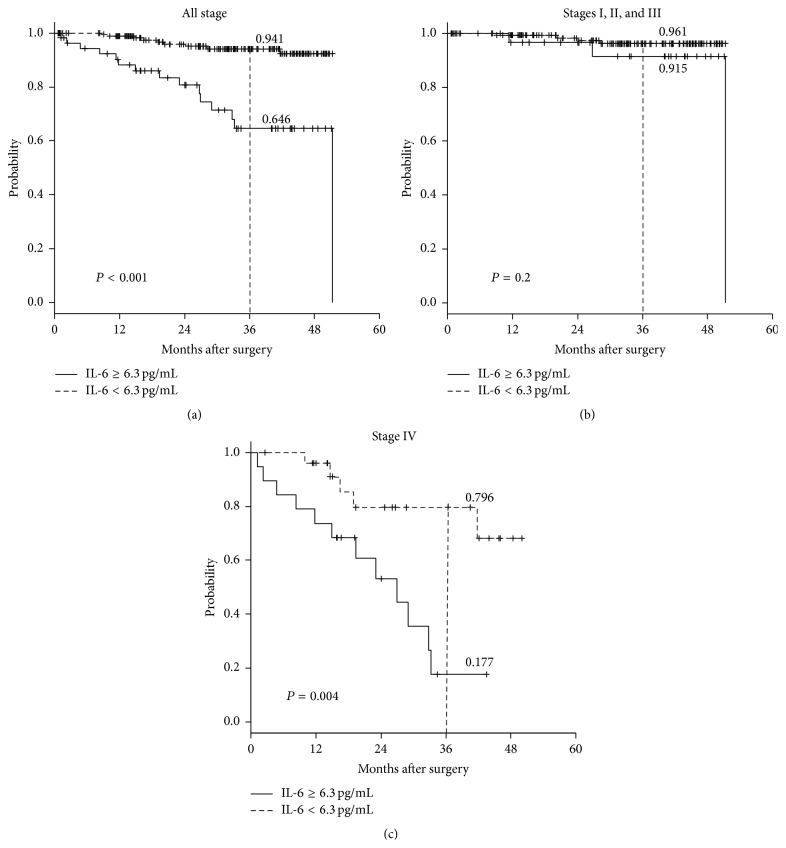
Three-year overall survival (OS) rates according to the preoperative serum interleukin-6 (IL-6) status (Kaplan-Meier analysis). (a) OS of all 233 patients. (b) OS of the 188 stages I–III colorectal cancer patients. (c) OS of the 45 stage IV patients.

During the follow-up period, 24 (10.0%) of the 233 patients died of cancer, and 1 of another, unrelated disease. Kaplan-Meier survival curves showed that patients with high IL-6 levels had a worse OS rate than those with low levels of IL-6 (*P* < 0.01) (Figure 2(a)). However, when only patients without synchronous metastatic lesions (stages I, II, and III) were analyzed, the OS rates were almost similar (*P* = 0.2) between patients with high and low IL-6 levels (Figure 2(b)). On the other hand, in stage IV patients, the OS rate of patients with high levels of IL-6 was significantly worse than that for those with low IL-6 levels (*P* = 0.004) (Figure 2(c)). Multivariate Cox hazard regression analysis revealed no independent factor associated with OS in stages I–III patients (data not shown).

#### 3.2.2. Disease-Free Survival

**Figure 3:**
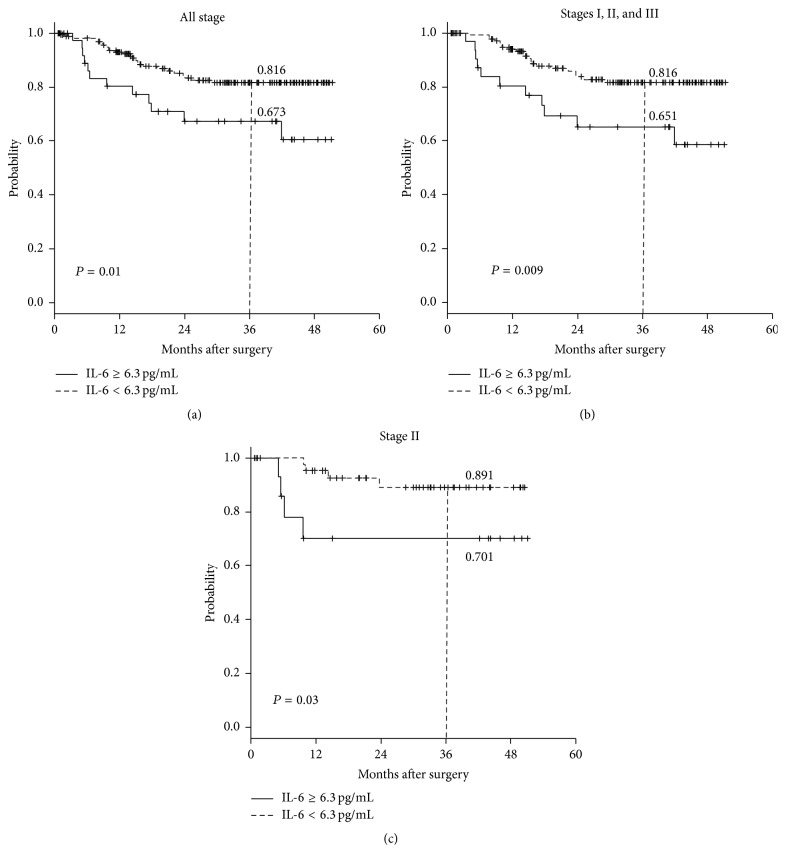
Disease-free survival (DFS) rates according to the preoperative serum interleukin-6 (IL-6) status. (a) DFS of all 207 patients who underwent curative resection. (b) DFS of 188 patients with stages I–III colorectal cancer. (c) DFS of the 60 stage II patients.

During follow-up, recurrence occurred in 37/233 patients (17.9%); among them, 12 of 37 patients had high preoperative IL-6 levels. The sites of metastasis were the liver, lung, and pelvis in 13, 10, and 5 patients, respectively. The DFS rate according to the serum IL-6 status was analyzed among the 207 CRC patients who underwent curative intent resection (Figure 3(a)). Patients with low serum levels of IL-6 had significantly longer DFS than those with high serum levels of IL-6 (*P* = 0.01). Similar differences were also observed after excluding stage IV patients from the evaluation, with patients classified as having TNM stages I–III disease who had low levels of serum IL-6 experiencing significantly longer DFS than those with high levels of serum IL-6 (*P* = 0.009) (Figure 3(b)). Similarly, when each stage was analyzed separately, a significant difference was also observed for TNM stage II patients (*P* = 0.03) (Figure 3(c)).

Based on this finding, we further investigated the correlations between each clinicopathological parameter and DFS by using the Cox proportional hazard model in TNM stage II patients (*n* = 60). The results revealed that high preoperative serum IL-6, high preoperative serum CRP, and pT4 disease were independent prognostic factors for stage II CRC patients (*P* = 0.01, *P* = 0.04, and *P* = 0.02, resp., Table 4). In this study, obstruction was not an independent prognostic factor (*P* = 0.4).

## 4. Discussion

Clinically, significant correlations between elevated serum IL-6 levels and other clinical factors such as serum CEA [14, 15], CRP [16, 17], and bowel obstruction [18, 19] have been previously reported. Further, it has also been reported that injections of CEA into murine models induced the release of cytokines, including IL-6, in vivo [20]. In addition, Gangopadhyay et al. reported that cytokines, such as IL-1*α*, IL-1*β*, IL-6, and tumor necrosis factor-*α* were induced by treating Kupffer cells with CEA in vitro [21]. These elevations have been hypothesized to be a causative factor of several cancers and to be related to prognosis [22, 23]. Taken together, these findings suggest a relationship between elevated serum IL-6 and CEA levels. Accordingly, our results also suggested that a high preoperative serum IL-6 level correlated with the levels of CEA and CRP and with colorectal obstruction.

On the other hand, a previous report demonstrated that endotoxin (lipopolysaccharide) production was induced by bowel obstruction. Endotoxins are found in the outer membrane of Gram-negative bacteria and are released by the destruction of the bacterial cell walls. They are known to cause various biological responses, including sepsis [18]. Furthermore, endotoxins activate inflammatory mediators such as IL-6 within the intestinal muscularis [19]. Hence, our results suggest that the high preoperative IL-6 levels may correlate with colorectal obstruction as a result of this relationship between endotoxins and IL-6 activation. However, in our study, serum endotoxin levels were not measured.

Furthermore, our results also showed a discrepancy regarding the order of the TNM stages and the IL-6 level. As shown in Figure 1(c), the mean IL-6 level of stage II patients was relatively higher than that of stage III patients. Similar results have been shown in several previous studies [24], but the reason for or the mechanism underlying this phenomenon is still not clear. However, the most important issue is that these patients with high IL-6 levels might have a poorer prognosis than patients with low levels of IL-6 do. The adaptation of adjuvant chemotherapy for stage II CRC remains controversial, and investigation of risk factors for recurrence in stage II CRC patients may help us to better design the appropriate adjuvant chemotherapy regimen. To date, depth of invasion (pT4); preoperative CEA level; lymphatic, venous, or perineural invasion; obstruction; and perforation have been reported as risk factors for recurrence in stage II CRC patients [25–27]. The recent American Society of Clinical Oncology guidelines recommend that adjuvant chemotherapy for stage II colon cancer patients should be determined based on the presence of the inadequately sampled lymph nodes, T4 disease, perforation, and poorly differentiated histology. Interestingly, many of these adverse factors correlated with high serum IL-6 levels in the present study, in which high preoperative serum IL-6 and low preoperative serum albumin were moreover found to be prognostic factors for stage II CRC patients, as determined using Cox proportional hazard models.

Previous studies have already reported a relationship between serum IL-6 levels and disease status in CRC patients [28–30]. Furthermore, Chung et al. reported that tissue expression of IL-6 may also represent a useful predictor of prognosis in CRC [31], and similar results have been reported in several different types of carcinoma [7–12].

In this study, patients with low preoperative levels of IL-6 experienced longer OS than those with higher levels of IL-6. However, we did not find a statistical difference in OS rates according to the serum IL-6 level. We believe that this lack of significance was likely a result of the shorter follow-up period in this study compared to that in other studies. On the other hand, we succeeded in showing that the DFS of patients with high IL-6 was significantly poorer than that of patients with lower IL-6, and we believe that the most important result of the present study was finding that high serum IL-6 was a risk factor for CRC recurrence, including stage II patients. In this study, obstruction did not significantly influence DFS in stage II patients, but the reason for this finding is unclear.

There are currently few reports investigating the correlation between IL-6 and DFS in CRC. In the present study, for all patients, and for stages I-III patients, low preoperative levels of IL-6 were significantly associated with a longer DFS; these results indicate that preoperative serum IL-6 is an independent risk factor for recurrence, and this information may aid in the decision-making regarding adjuvant chemotherapy.

## 5. Conclusions

Preoperative serum IL-6 influences CRC recurrence. Importantly, this result also applies to stage II cancer patients, and this finding may aid in the decision-making regarding adjuvant therapy in these patients.

## Figures and Tables

**Table 1:** Characteristic of the study patients (*n* = 233).

	Number of patients	Mean values (±SD)	%
IL-6 (pg/mL)			
Colorectal cancer	233	6.6 (±9.9)	94.7
Healthy control	13	2.6 (±2.5)	5.3
Age (years)		69.1 (±10.2)	
BMI		22.2 (±3.8)	
Albumin (g/dL)		4.0 (±0.5)	
CRP (mg/dL)		0.5 (±1.0)	
CEA (ng/mL)		54.6 (±451.6)	
CA19-9 (U/mL)		66.1 (±344.1)	
VEGF (pg/mL)		92.9 (±128.5)	
Tumor location			
Rectum	78		33.5
Colon	155		66.5
TNM classification			
0	13		5.6
1	52		22.3
2	60		25.8
3	63		27.0
4	45		19.3

IL-6: interleukin-6; BMI: body mass index; CRP: C-reactive protein; CEA: carcinoembryonic antigen; CA19-9: carbohydrate antigen 19-9; VEGF: vascular endothelial growth factor.

**Table 2:** Results of the univariate analyses (*n* = 233).

		High IL-6 levels (≥6.3 pg/mL) *n* = 57 (%)	Low IL-6 levels (<6.3 pg/mL) *n* = 176 (%)	*P* value
Age (years)	≥75	24 (32.4)	50 (67.6)	0.07
	<75	33 (20.8)	126 (79.2)	
Sex	Female	20 (21.3)	74 (78.7)	0.44
	Male	37 (26.6)	102 (73.4)	
BMI	≥25	10 (19.2)	42 (80.8)	0.37
	<25	47 (26.0)	134 (74.0)	
Albumin (g/dL)	≥4.0	19 (14.4)	113 (85.6)	<0.01
	<4.0	38 (37.6)	63 (62.4)	
CRP (mg/dL)	≥0.5	34 (63.0)	20 (37.0)	<0.01
	<0.5	23 (12.8)	156 (87.2)	
CEA (ng/mL)	≥5.0	30 (32.3)	63 (67.7)	0.03
	<5.0	27 (19.3)	113 (80.7)	
CA19-9 (U/mL)	≥37.0	19 (38.0)	31 (62.0)	0.02
	<37.0	38 (20.8)	145 (79.2)	
VEGF (pg/mL)	≥145	12 (30.0)	28 (70.0)	0.42
	<145	45 (23.3)	148 (76.7)	
Tumor location	Right	24 (30.4)	55 (69.6)	0.15
	Left	33 (21.4)	121 (78.6)	
Pathological findings				
Differentiation	Poor	6 (42.9)	8 (57.1)	0.11
	Others	51 (23.3)	168 (76.7)	
Lymphatic invasion	Positive	48 (26.4)	134 (73.6)	0.27
	Negative	9 (17.6)	42 (82.4)	
Venous invasion	Positive	50 (29.9)	117 (70.1)	<0.01
	Negative	7 (10.6)	59 (89.4)	
TNM classification				
T	<4	28 (16.3)	144 (83.7)	<0.01
	4	29 (47.5)	32 (52.5)	
N	Positive	31 (30.4)	71 (69.6)	0.07
	Negative	26 (19.8)	105 (80.2)	
M	Positive	19 (42.2)	26 (57.8)	<0.01
	Negative	28 (15.7)	150 (84.3)	
Liver metastasis	Positive	11 (37.9)	18 (62.1)	0.27
	Negative	46 (22.5)	158 (77.5)	
Obstruction	Positive	23 (57.5)	17 (42.5)	<0.01
	Negative	34 (17.6)	159 (82.4)	

The analyses were performed using Fisher's exact test. The results are presented as the number of patients.

IL-6: interleukin-6; BMI: body mass index; CRP: C-reactive protein; CEA: carcinoembryonic antigen; CA19-9: carbohydrate antigen 19-9; VEGF: vascular endothelial growth factor.

**Table 3:** Logistic multivariate analysis for the associations with interleukin-6 for all patients (*n* = 233).

	OR	95% CI	*P* value
CRP	2.270	1.100–4.690	<0.01
CEA	9.530	4.490–20.200	0.04
Obstruction	0.255	0.110–0.595	<0.01
T4	0.446	0.207–0.960	0.04

OR: odds ratio; CI: confidence interval; CRP: C-reactive protein; CEA: carcinoembryonic antigen.

**Table 4:** Results of the multivariate Cox hazard model for disease-free survival in stage II colorectal patients (*n* = 60).

	Hazard ratio	95% CI	*P* value
CEA	5.1	0.9–30.6	0.07
CRP	8.2	1.1–61.2	0.04
IL-6	0.1	0.01–0.6	0.01
Obstruction	2.0	0.4–10.2	0.4
T4	6.7	1.4–31.6	0.02

OR: odds ratio; CI: confidence interval; CEA: carcinoembryonic antigen; CRP: C-reactive protein; IL-6: interleukin-6.
